# Physicians’ legal knowledge of informed consent and confidentiality. A cross-sectional study

**DOI:** 10.1186/s12910-022-00835-3

**Published:** 2022-09-16

**Authors:** Maria Cristina Plaiasu, Dragos Ovidiu Alexandru, Codrut Andrei Nanu

**Affiliations:** 1 Doctoral School, University of Medicine and Pharmacy of Craiova, 2 Petru Rares St, 200349 Craiova, Romania; 2 Department of Medical Informatics and Biostatistics, University of Medicine and Pharmacy of Craiova, 2 Petru Rares St, 200349 Craiova, Romania; 3 Department No. 14 of Orthopedics, Anesthesia and Intensive Care, University of Medicine and Pharmacy “Carol Davila”, 37 Dionisie Lupu St., Sector 2, 020021 Bucharest, Romania

**Keywords:** Legal knowledge, Informed consent, Confidentiality, Romanian physicians, Legal compliance, Legislation awareness

## Abstract

**Background:**

Only a few studies have been conducted to assess physicians’ knowledge of legal standards. Nevertheless, prior research has demonstrated a dearth of medical law knowledge. Our study explored physicians’ awareness of legal provisions concerning informed consent and confidentiality, which are essential components of the physician-patient relationship of trust.

**Methods:**

A cross-sectional study assessed attending physicians’ legal knowledge of informed consent and confidentiality regulations. The study was conducted in nine hospitals in Dolj County, Romania. Physicians were given a questionnaire with ten scenarios and instructed to select the response that best reflected their practice. We assessed the responses of physicians who claimed their practice to be entirely legal. Their legal knowledge was evaluated by comparing their answers to applicable laws. We also calculated a score for the physicians who admitted to committing a legal breach.

**Results:**

Of the 305 respondents, 275 declared they never committed any law violation. However, their median correct answer score was 5.35 ± 1.66 out of 10. The specialty was the strongest predictor of legal knowledge, with emergency physicians rating the lowest and non-surgical physicians scoring the highest. Physicians who worked in both private and public sectors were better knowledgeable about legal issues than those who worked exclusively in the public sector. Results indicate that physicians are aware of the patient’s right to informed consent but lack comprehensive understanding. While most physicians correctly answered simple questions, only a tiny minority identified the correct solution when confronted with ethical dilemmas. The physicians who acknowledged breaching the law, on the other hand, had a slightly higher knowledge score at 5.45 ± 2.18.

**Conclusion:**

Legal compliance remains relatively low due to insufficient legal awareness. Physicians display limited awareness of legal requirements governing patient autonomy, confidentiality, and access to health data. Law should be taught in all medical schools, including undergraduate programs, to increase physicians’ legal knowledge and compliance.

**Supplementary Information:**

The online version contains supplementary material available at 10.1186/s12910-022-00835-3.

## Introduction

The presumption of legal knowledge is a jurisprudential postulate indicating that individuals are assumed to know the law. Although ignorance of the law is not punishable, it may result in sanctions if it manifests as illegal conduct. Fundamentally, even if the state does not compel legal knowledge, it does impose conformity with the law. On the other hand, conformity does not always entail legal knowledge but may derive from personal convictions or serendipitous alignments [1]. Nevertheless, knowledge of the law is a prerequisite for ensuring compliance and solving ethical dilemmas [2].

Even though the law should generally be accessible to everyone, physicians may encounter various access and content accessibility obstacles. In several countries, medical education has included the study of law for some years to overcome these barriers. The primary objective is to educate physicians on the legal framework and better prepare them to deal with anticipated legal dilemmas. Contemporary medicine expects physicians to perform at a higher level than experienced practitioners and go beyond the bare minimum of a level of care. Physicians must incorporate legal knowledge into their clinical practice on an equal footing with scientific techniques in disease etiology and treatment [3]. Although integrating ethics and legal education into medical curricula is not a simple endeavor, numerous universities are currently undertaking it.

Only a few studies have been conducted to assess physicians’ knowledge of legal standards. Nevertheless, prior research has demonstrated a dearth of medical law knowledge among physicians. According to Skiba et al., Australian physicians are insufficiently aware of their legal responsibilities regarding informed consent. The study indicates that physicians are unaware of the court order requiring them to inform patients of any problem they may attribute significance to [4]. Riley’s research discovered that despite physicians’ attitude in favor of confidential treatment for adolescent patients, nearly half of the respondents were ignorant of the Michigan legislation governing a teen’s right to consent to confidential therapy [5]. Al-Busaidi et al. concluded that the majority of respondents were uninformed of the Omani medical laws applied to the sharing of confidential information with third parties [6].

There is no previous study to assess legal knowledge in Romania, but some previous research indicated low legal compliance among Romanian physicians [7]. Although prior research indicated that most physicians’ practices contravened the legal criteria for informed consent and confidentiality, it did not investigate the causes. As physicians’ behavior may be influenced not only by their legal knowledge, but also by a variety of other factors, such as insufficient training, lack of time, laziness, and hospital habits [8] we believe it is important to assess physicians’ awareness of the laws governing patient rights.

Our study offers an insight into physicians’ legal knowledge in a post-communist country with relatively new and inconsistent medical legislation. Due to the essential role in the physician-patient relationship of trust, we decided to explore physicians’ legal knowledge of informed consent and confidentiality. Physicians’ awareness and compliance in this sector are the most important since patients lack the knowledge and power to participate in the shared decision-making [9].

We researched physicians’ awareness of the regulations regarding the duties related to informed consent and confidentiality as stipulated by the Romanian medical legislation (Law number 46/2013, Law number 85/2016, and Minister of Health Order number 1411/2016). Since Romania ratified the European Convention from Oviedo and subsequently joined the European Union, the rights of Romanian patients are consistent with those guaranteed by the European Union’s legislation. According to the law, physicians are required to inform patients before performing any medical act and acquire their written consent for all procedures that may pose risks. The law also acknowledges that patients with decision-making capacity have full decisional autonomy, whereas a medical arbitration commission has the final authority to determine the appropriate therapy for patients without decision-making capacity. In an emergency, physicians are compelled to intervene without the consent of a proxy. Additionally, physicians are required to maintain confidentiality with third parties and provide complete information to patients.

In the past, bioethics addressed these issues, leading to the regulation of patient rights. As a result, physicians are now required by law to perform specific duties, and failure to do so may result in civil or criminal sanctions. Since current Romanian attending physicians had received no formal education in law throughout their undergraduate studies, we anticipated a lack of legal understanding. In 2017, the University of Medicine and Pharmacy of Bucharest included the study of legal components pertinent to patients’ rights and medical malpractice, and it is the only university in the country to have done so. Consequently, no current resident or attending Romanian physician has studied medical law.

## Materials and methods

### Setting

We conducted a cross-sectional study to determine attending physicians’ understanding of laws regarding informed consent and confidentiality. The study was conducted in nine hospitals in Dolj County, Romania between November 2021 and March 2022. Dolj county is located in the southwestern part of Romania, with over 700,000 residents (7th place among Romanian counties) and a monthly net income of approximately 630 euros (16th place in Romanian counties). The local healthcare system includes thirteen public hospitals (one of which is a psychiatric facility that was excluded from the study due to different legislation applicable to patients). The healthcare system in Romania is built on a modified version of the Bismark model, and although it includes both public and private facilities, it remains highly centralized. Because resources and employees are primarily concentrated in the public sector, we focused our research on the public hospitals in the region. Six out of the twelve public hospitals agreed to our invitation to participate in the study. We also sent invitations to the three private hospitals with the largest number of physicians in the region, of which two participated in the study. To ensure a diverse range of healthcare practices and county coverage, we included different local hospitals: one tertiary university hospital, one secondary community hospital, two specialty hospitals, two primary hospitals, and two private day hospitals.

### Participants and data collection

The study was conducted on a convenience sample consisting of physicians working in hospital settings from Dolj county on a voluntary base. To approach the participants, we sought administrative consent from the hospital management. On the basis of their affirmative responses, we approached the hospital ward heads to seek second approval for each ward. Due to the pandemic, the ward heads deposited printed copies of the questionnaire in on-call rooms, where physicians could easily access them. After a week or two, depending on the circumstances, we collected all of the filled-in questionnaires. We have distributed 418 printed questionnaires in accordance with the information provided by the hospital ward heads. We included all available attending physicians. However, psychiatric physicians were not included due to their distinct legal responsibilities. Additionally, physicians who worked solely in laboratories and had no contact with patients were also excluded from the cohort. Considering the particularities and similarities across specialties, we opted to group physicians into five categories: surgical specialties (cardiovascular, general, oral and maxillofacial, pediatric, plastic, thoracic, neurosurgery, ophthalmology, orthopedic, otolaryngology, urology), non-surgical specialties (allergy and immunology, dermatology, gastroenterology, hematology, internal medicine, neonatology, neurology, nephrology, radiotherapy, pediatrics, physical medicine and rehabilitation, pulmonology, rheumatology, oncology), obstetrics-gynecology, emergency, and anesthesia and intensive care.

### Questionnaire

The questionnaire was adapted from one previously published by Nanu et al. [7] in light of the findings of the previously cited research. We added three more scenarios regarding informed consent and one self-assessment question to the initial questionnaire, validated in 2007. All additional questions were validated by a panel of five experts. We asked the experts to evaluate if the additional questions were essential, relevant, and intelligibly formulated. After calculating the index of content validity and ratio, we retained all additional questions.

We divided the questionnaire into three sections:

Section 1: Information on the research participants concerning medical specialty, age, years in practice, workplace sector, and location.

Section 2: Multiple choice scenario-based questions on informed consent and confidentiality (n = 10 questions). We provided physicians with ten distinct scenarios, seven of them referring to informed consent and three to confidentiality. To each scenario, we offered three alternative answers, only one of which was in accordance with the law. We instructed the participants to choose the responses that most accurately reflected their regular practice. Respondent’s knowledge of informed consent was tested by scenarios number 3, 4, 5, 6, 7, 9, and 10, while confidentiality knowledge was addressed by questions number 1, 2, and 8.

Section 3: Self-assessment (n = 1 question). Finally, we asked them to self-assess their practice and respond to the question of whether they engaged in any medical acts that could be considered a breach of medical laws in the preceding 3 years.

The questionnaire was applied in the Romanian language to the Romanian physicians. An additional file presents the English translation of the questionnaire in more detail (see Additional file 1).

### Data analysis

The question asking physicians to self-assess their compliance with the law also represented the exclusion criteria. To calculate physicians’ legal knowledge ratings, we included those respondents who answered negatively to the self-assessment question and declared that they did not engage in potentially illegal medical acts. Their responses to the ten scenario-based questions were compared to the applicable legal standards. The correct answer was the legally acceptable one, and each scenario-based question was weighted equally (one point for each correct answer and zero points for incorrect answers). Additionally, we calculated the compliance score for the physicians who admitted to having committed a legal breach and compared the results. We decided that a high Kuder–Richardson Reliability Coefficient was not desirable, as the study relates to education knowledge, and each question evaluates an aspect on an entirely different scale, loading onto different factors [10].

We used Microsoft Excel (Microsoft Corp., Redmond, WA, USA), together with the XLSTAT add-on for MS Excel (Addinsoft SARL, Paris, France) and IBM SPSS Statistics 20.0 (IBM Corporation, Armonk, NY, USA) for processing the data. Descriptive analysis of the study group was performed with MS Excel. Normality tests (Anderson-Darling) and complex statistical tests (Chi-Square, Kruskal–Wallis, Friedman, etc.) were performed using the XLSTAT add-on or SPSS. We used the Anderson-Darling test to verify the normality of the data. None of the numerical variables investigated had a normal distribution of data, globally or inside each studied group. Because the study involved numerical comparisons between more than two groups that did not have a standard (Gaussian) distribution, the nonparametric Kruskal-Wallis test was primarily used, followed by a posthoc analysis using the Dunn method for multiple pairwise comparisons with Bonferroni correction. Categorical data were compared using the Chi-square test (χ^2^), which is a statistical test that shows if there is a connection (association or influence) between two factors. It was used to interpret incidence tables generated by cross-tabulation of two categorical variables recorded in the study.

## Results

305 out of the 418 questionnaires sent out to attending physicians were returned resulting in a response rate of 72%. All the 305 questionnaires were completely filled in. To calculate the legal knowledge score, we excluded the 31 physicians who participated in the poll but said they had engaged in medical practices that might be considered unlawful. Among the remaining 274 clinicians who indicated they had committed no illegal medical act, 87% were under 60 years old, and 50% had worked for less than 15 years. Table 1 summarizes the cohort’s demographic characteristics, including physicians’ specialty, work sector, age, and years in practice. The average age of physicians was 43.2 ± 10.9 years, and the average number of years in practice was 16 ± 10.8.

**Table 1:** Physicians’general characteristics

n = 274	n	%
*Groups*		
Anesth. intensive care	17	6.2
Surgical	54	19.7
Non-surgical	131	47.8
Obstetrics gynecology	56	20.4
*Emergency*	16	5.8
Work sector		
Private	14	5.1
Public	137	50
Both	123	44.9
*Age*		
< 30	26	9.4
30–39	85	31
40–49	77	28.1
50–59	51	18.6
> 60	23	8.3
Unknown	13	4.7
*Years in practice*		
1–5	56	20.4
6–10	37	13.5
11–15	46	16.7
16–20	34	12.4
21–25	36	13.1
26–30	26	9.4
> 30	23	8.3
Unknown	16	5.8

The median correct answer score in the ten scenario-based questions was 5.35 ± 1.66 out of 10. The specialty was the strongest predictor of legal knowledge, with emergency physicians rating the lowest and non-surgical physicians scoring the highest. Dunn’s post hoc test with Bonferroni correction revealed significant differences (*p* < 0.001) between emergency physicians and surgeons, non-surgery physicians, and obstetrics-gynecology physicians, but not between emergency physicians and anesthesia or intensive care physicians (*p* < 0.0235) (Table 2).

**Table 2:** Physicians’ Median Knowledge and Standard Deviation total, informed consent, and confidentiality scores

Specialty	Total n* = 10	Informed consent n = 7	Confidentiality n = 3
	Mean (SD)	Mean (SD)	Mean (SD)
Anaesth. Intensive Care	4.94 ± 1.64	3.82 ± 1.29	1.12 ± 0.99
Surgical	5.44 ± 1.55	4.02 ± 1.16	1.43 ± 0.77
Non-surgical	5.58 ± 1.64	3.92 ± 1.17	1.66 ± 0.83
ObGyn	5.45 ± 1.48	3.88 ± 1.08	1.57 ± 0.91
Emergency	3.31 ± 1.66	2.19 ± 1.17	1.13 ± 0.62
TOTAL	5.35 ± 1.66	3.82 ± 1.22	1.53 ± 0.85

*Number of scenarios equals total score

The analysis of additional variables revealed that physicians who worked in both private and public sectors were more knowledgeable about legal issues than those who worked exclusively in the public sector (*p* < 0,005). On the other hand, our research indicated that respondents’ age and the number of years in practice had no impact on their legal knowledge (Table 3).

**Table 3:** Physicians’ Mean Score and Standard deviation detailed according to demographics

Demographics	Total n* = 10	Informed consent n = 7	Confidentiality n = 3
	Mean (SD)	Mean (SD)	Mean (SD)
*Age*			
< 30	4.69 ± 1.52	3.35 ± 1.20	1.35 ± 0.69
30–39	5.64 ± 1.63	4.07 ± 1.22	1.56 ± 0.82
40–49	5.38 ± 1.75	3.93 ± 1.31	1.45 ± 0.89
50–59	5.37 ± 1.81	3.81 ± 1.25	1.56 ± 0.93
> 60	6.40 ± 1.67	4.40 ± 1.67	2.00 ± 0.00
Unknown	5.43 ± 1.40	3.71 ± 0.95	1.71 ± 0.49
*Years in practice*			
1–5	5.14 ± 1.58	3.70 ± 1.17	1.45 ± 0.81
6–10	5.65 ± 1.69	3.97 ± 1.17	1.68 ± 0.91
11–15	5.46 ± 1.77	3.93 ± 1.39	1.52 ± 0.81
16–20	5.41 ± 1,65	4.00 ± 1.23	1.41 ± 0.86
21–25	5.42 ± 1.61	3.72 ± 1.16	1.69 ± 0.82
26–30	5.73 ± 1.85	4.15 ± 1.32	1.58 ± 0.86
> 30	4.70 ± 1.46	3.26 ± 1.01	1.43 ± 0.90
Unknown	5.19 ± 1.68	3.75 ± 1.13	1.44 ± 0.96
*Work sector*			
Both	5.65 ± 1.66	4.02 ± 1.19	1.63 ± 0.93
Private	5.64 ± 1.34	4.14 ± 1.17	1.50 ± 0.52
Public	5.06 ± 1.65	3.62 ± 1.23	1.44 ± 0.79

*Number of scenarios which equals total score available

### Knowledge of informed consent legislation

The median correct answer score in the seven scenario-based questions regarding informed consent was 3.82 ± 1.22 out of 7. We discovered disparities in physicians’ knowledge of informed consent legislation across specialties. Surgeons were more knowledgeable of informed consent regulations, whereas emergency physicians were the least familiar. Additionally, Dunn’s post hoc test with Bonferroni correction revealed significant differences (*p* < 0.001) between emergency physicians and the other specialties (Table 2).

#### Scenarios pertaining a patient with medical decision-making capacity

Most physicians (98.9%) declared they always informed the patient in detail before a potentially risky maneuver. However, fewer physicians (94.16%) were knowledgeable that written consent from the patient is mandatory. Even fewer physicians (55.11%) knew they should seek written permission from patients before collecting and examining biological samples.

Once confronted with an ethical dilemma, most physicians offered the incorrect response. When asked to imagine a hypothetical life-threatening scenario in which a patient declines emergency assistance, just 32.48% of respondents recognized the law required them to accept the patient’s decision.

In another scenario, 70.8% of physicians believed they had the right to terminate a patient’s therapeutic relationship if the patient refused to adhere to the prescribed therapy.

#### Scenarios pertaining a patient without medical decision-making capacity

A slight majority (52.19%) knew that if an incompetent patient’s relatives refused to consent to proper medical care, the legislation required an arbitrary medical committee to select the most appropriate treatment for the patient.

Additionally, only one of the sixteen emergency physicians rightly indicated that when physicians intervene in an emergency without the consent of a substitute decision-maker, they are legally required to document the circumstance. The total percentage of respondent physicians correctly answering the question was low (32.12%).

Between emergency physicians and the other groups, there were statistically significant differences.

### Knowledge of conconfidentialityd health data disclosure legislation

The median correct answer score in the three scenario-based questions was 1.53 ± 0.85 out of 3. Two questions addressed the issue of disclosing information about patients’ health to third parties, close relatives included. We asked physicians whether health data could be disclosed to third parties in the first question. We offered as an alternative the option of obtaining the patient’s consent prior to disclosure. Most physicians (70.8%) selected the correct response. There were differences between specialties; for example, only one in two anesthesia and intensive care (47.06%) and emergency physicians (50%) knew the information about a patient’s health was confidential. In the following case, we asked physicians if a patient’s treatment details could be divulged to third parties. This question did not include the patient’s permission as a viable response. Only 31.75% of respondents knew that the law prohibited disclosure in this circumstance.

In the scenario centered on physicians’ attitudes regarding sharing health information with patients, almost half of respondents (49.64%) were unaware that patients had the right to acquire complete health records.

The confidentiality general knowledge score was not influenced by physicians’ specialty, age, years in practice, or work sector (Table 3).

### Physicians who declared legal breaches

In terms of demographic features and legal compliance, there was no statistically significant difference between physicians who admitted committing a law violation and those who did not.

In this group, the average age of physicians was 41.6 ± 7.5 years, and the average number of years in practice was 14.7 ± 8.1. Within this group, there was no significant variance in legal compliance attitude based on specialty, work sector, age, or the number of years in practice. The compliance score for these responders was 5.45 ± 2.18.

## Discussion

A deeper understanding of medical legislation is critical since it significantly impacts legal compliance and consequently the integrity of the patients’ rights. Non-compliance with the law may expose physicians to significant risks in the event of malpractice claims.

Our study indicates that physicians have insufficient knowledge of informed consent and confidentiality laws. Compared to other studies regarding physicians’ knowledge of various aspects of the law, our respondents’ overall score was marginally lower [11]. The confidentiality score of 1.53 out of 3 is consistent with Karasneh’s (7 out of 14) but lower than Beltran Aroca’s (6.8 out of 10) and Tegegne’s (3.91 out of 7) [8, 12, 13].

Our findings corroborated prior studies indicating that although specialty was a predictor of legal awareness, age and years of practice were not [14–16]. While we cannot explain the disparity in legal knowledge within specialties, a plausible explanation for the homogeneity of knowledge scores across age and years of practice groups is the abiding absence of legal or ethical education from university curricula. Additionally, we identified a gap between physicians who work entirely in the public sector and those who work in both the public and private sectors, implying that the private sector exposes physicians to additional legal standards. This could be attributed to the fact that private-sector Romanian physicians are more prone to be accused of medical malpractice, according to a recent study [17]. Results indicate that physicians are aware of the patient’s right to informed consent but lack comprehensive understanding. Informed consent is a complex process. It is more than obtaining patients’ signatures on a consent form [18]. While physicians are aware of their duties to inform, obtain and document consent, they demonstrate low legal understanding in more complex scenarios.

According to the law, physicians ought to be aware of the regulations regarding the respect for the right to autonomy, which include the obligation to inform the patient prior to any medical procedure, to obtain written permission for medical procedures that involve risks, to respect the decision of patients with decision-making capacity, and to make the appropriate medical decision for patients without decision-making capacity. In addition, physicians should be aware of their obligation to respect patient confidentiality and to grant patients complete access to their medical data.

In essence, physicians’ responses suggest a fundamental incomprehension of the core idea of informed consent, which includes the patients’ right to accept or refuse any medical procedure or treatment according to their beliefs and values without jeopardizing the patient-doctor relationship. For instance, there is a prevalent misconception among physicians that they are permitted to end their relationship with a patient in the event of treatment refusal. A previous survey found a better result, with slightly more than half of the family physicians responding that they would not discontinue therapy if patients declined their consultation request [11]. In direct contrast to our findings, Craig’s research revealed that 98.7% of respondents acknowledged a patient’s right to refuse treatment [19].

Our research identified more situations where physicians’ insufficient legal awareness resulted in arbitrary decision-making and low legal compliance. In one such example, roughly a third of the physicians acknowledged a patient’s refusal to undergo potentially life-saving surgery. The low compliance rate matches White’s study (32%), which presented physicians with a similar scenario [20]. Although White’s findings support the theory that there is a correlation between legal knowledge and legal compliance, he argues that there may be additional grounds for low legal compliance in the case of a life-threatening event. Moreover, he relates it to ethical considerations. According to his findings, the physicians’ appraisal systems are built on a hierarchy of decision-making criteria, in which legislation is subordinate to clinical variables connected to the patient’s health [20, 21]. Craig’s study partially supports those findings, implying that physicians occasionally evaluate a patient’s motivation and disregard his decision accordingly if they have different viewpoints or values [19]. His research, however, shows that after receiving educational training, physicians demonstrate higher levels of legal compliance. According to our findings, physicians are more predisposed to make medical rather than ethical decisions when ignorant of applicable laws. Nonetheless, we believe that additional research is required in order to gain a comprehensive understanding of the topic.

Furthermore, the analysis of responses regarding patients without decision-making capacity indicated that physicians have a limited understanding of the applicable laws, both in emergency and non-emergency scenarios. According to Romanian legislation, a substitute decision-maker must be found if a patient lacks the legal capacity to make medical decisions. A patient’s substitute can be a parent, spouse, family member, or a conventional or court-appointed representative. They may seek and accept medical interventions for the patient but cannot veto a necessary medical procedure. Nearly half of the respondents in this instance were unaware that they were legally required to request an arbitrary committee decision. Additionally, physicians had little knowledge of their legal duty to complete a report when unable to obtain consent from a substitute decision-maker due to an emergency.

Moreover, the average confidentiality score indicates an insufficient awareness of the minimal legal standards for disclosing health information to third parties and assuring the patients’ complete access to their data. Despite earlier research indicating that specialty [22], age, and years in practice [8, 12], are predictors of confidentiality breaches, our data revealed no variations among physicians. However, we did not assess other variables considered in prior research, such as gender, ethics education, patient volume, and the frequency of ethical issues physicians encounter in their field of practice [13].

Previous studies revealed multiple causes for confidentiality violations. Some are the product of negligence, and others result from poor infrastructure [22], while others are the consequence of legislation unawareness. In our study, physicians demonstrated some unfamiliarity with legal requirements. On the good side, data indicate that most physicians know they may disclose confidential information to third parties with their patients’ express permission. Nonetheless, when unaware of the patient’s attitude, a sizable majority believed the law permits disclosure of health data.

Additionally, we noticed paternalistic behavior in supplying patients with access to their health records. A sizable proportion of respondents incorrectly assumed they were legally required to provide only minimal diagnostic and treatment information to patients.

We noticed a substantial discrepancy between our findings and other previously cited studies, especially those from Spain and Australia. We believe this is a result of several circumstances, including a relatively new Romanian medical legislation, a large number of laws, and the social and political context.

Comparing the two groups of physicians, those who have admitted to breaking the law and those who have not, we observed no significant differences in their interactions with patients. One of the plausible explanations could be attributed to the minority’s urge to adhere to the attitude of the majority, as identified in prior research [23].

## Limitations of the study

The primary study’s limitations originate from biases imposed by our method of assessing legal knowledge. First, when physicians’ opinions and laws align, compliance with the law may not result from actual knowledge. Second, self-perception biases might have influenced physicians to assess their practice as legal since individuals frequently convince themselves that their inadvertent unethical actions are legitimate [24].

Additionally, there are some limitations due to the scarcity of physicians in emergency, anesthesia, and intensive care units. Furthermore, despite our best efforts, we could not conduct our research in several tertiary hospital wards due to hospital ward heads’ reluctance. Finally, our respondents were selected from a single county, and, despite the homogeneity in national educational curricula, the results may not be generalizable to all regions.

## Conclusion

Legal compliance remains relatively low due to insufficient legal awareness. Although physicians assess their activity as lawful, almost half of the cases resulted in violations of legal requirements. Physicians struggle with both procedural legal requirements and substantive aspects of the law. In our experience, physicians display limited awareness of patient autonomy and bodily integrity and hence are not fully capable of acknowledging patients’ physical boundaries as sovereign [25]. Additionally, physicians are insufficiently aware of their duties regarding patients’ confidentiality and health data access.

Our study supports previous research [26–29], indicating legal education should be increased among physicians. Law should be taught at all medical schools, including undergraduate programs, to increase physicians’ legal knowledge and compliance. Integrating legal education into medical curricula is not a simple task, but immediate interventions should be taken where an insufficient level of compliance is identified.

## Supplementary Information

**Additional file 1. **Questionnaire on informed consent and confidentiality.

## Data Availability

Data and materials can be found at the corresponding author. Although we cannot make it public because it contains information that could compromise health care facilities’ privacy, it may be made available upon reasonable requests. Furthermore, while we did not collect personal information, employers may be able to identify responders through demographic data correlations.

## Abbreviations

Anaesth. Intensive Care: Anaesthesia and intensive care
ObGyn: Obstetrics–gynecology
