# Supplementary material for: Physicians’ legal knowledge of informed consent and confidentiality. A cross-sectional study
Source: BMC Med Ethics. 2022 Sep 16;23:93. doi: 10.1186/s12910-022-00835-3 (PMC9479386; doi:10.1186/s12910-022-00835-3)
Supplement: Supplementary file 1 — Additional file 1. Questionnaire on informed consent and confidentiality. [file 12910_2022_835_MOESM1_ESM.docx]

Questionnaire / Correct answers

(Translated into English)

The purpose of the research is to identify the main vulnerabilities of medical practice in the face of potential malpractice accusations, in order to reduce professional risks and avoid situations of medical malpractice. Please answer the questions accordingly to your practice.

Your answers are completely anonymous and we do not collect, in any way, personal data.

I agree

I declare to be an attending physician and that I have not previously filed this questionnaire: a) Yes, b) No

Section 1: Information on the research participants:

1. Years in practice: ____
2. Age: _____
3. Medical specialty: surgical, nonsurgical, obstetrics-gynecology, emergency, anesthesia, and intensive care.
4. Workplace sector: public, private, both
5. Location: _______

Question no. 1: Is the information about the health state of a patient with decision-making capacity communicated to thirds?” Proposed answer options: a) Yes, only to close relatives. b) No. c) Yes, with the express acceptance of the patient.

*The correct answer envisaged: “Yes, with the express acceptance of the patient” (c).*

Question no. 2: “Is the information about the treatment followed by a patient communicated to thirds?” Proposed answer options: a) Yes if they accompany the patient to hospital admission, b) Yes, to the patient’s family members. c) No.

*The correct answer envisaged: “No” (c).*

Question no. 3 “Biological samples are collected and tested:” Proposed answer options: a) Based on the doctor’s decision, exclusively. b) After acquiring the patient’s consent. c) After consulting the management of the healthcare unit, based on medical and financial criteria.

*The correct answer envisaged: “After acquiring the patient’s consent” (b)*

Question no. 4: “You are about to perform a potentially risky procedure on the patient. Do you explain the risks to the patient?” Proposed answer options: a) Yes, always, in detail. b) No, to protect the patient. c) No, the decision made exclusively belongs to the doctor.

*The correct answer envisaged: “Yes, always, in detail” (a).*

Question no. 5: „In case of a life-threatening emergency, if the patient refuses the intervention, how can you proceed?” Proposed answer options: a) I obtain consent from close relatives. b) the decision is made by a medical arbitration commission. c) I respect the patient’s decision.

*The correct answer envisaged: I respect the patient’s decision (c)*

Question no. 6: “Have you obtained the written consent of the patients for risky procedures?” Proposed answer options: a) No, this decision belongs to the doctor.

b) Yes, always. c) Only when it involves major risks.

*The correct answer envisaged: “Yes, always” (b).*

Question no. 7: „ If the patient lack decision-making capacity and the patient’s relatives refuse to agree to a potentially risky medical procedure. How can you proceed?” Proposed answer options: a) I act in the patient’s best interest. b) I respect the patient relatives' decision. c) I decline the decision to a medical arbitration commission constituted at the hospital level.

*The correct answer envisaged: “ I decline the decision to a medical arbitration commission constitute at the hospital level.” (c).*

Question no. 8: “Has the patient access to his/her medical information about the investigations performed, diagnostic and recommended treatment?” Proposed answer options: a) Not necessary; when the patient is released from the hospital, he/she is informed about the treatment scheme and the necessity of coming back for medical checks. b) Yes, always, completely. c) Only information about the diagnostic and treatment methods is

made available to the patient.

*The correct answer envisaged: “Yes, always, completely” (b).*

Question no.9: “The patient cannot express his/her consent, and his/her health condition requires emergency procedures. The patient’s consent file is substituted by:”

Proposed answer options: a) The patient’s consent is not necessary for emergency situations. b) A written report, produced by the doctor who provides the medical care, attached to the patient’s consultation sheet. c) The decision is made by the board of the medical unit.

*The correct answer envisaged: “A written report, produced by the doctor who provides the medical care, attached to the patient’s consultation sheet” (b)*

Question no. 10: „ You may terminate the therapeutical relationship with one of your patients:” Proposed answer options: a) after you’ve accepted him/her as a patient, the relationship may be terminated only when the patient is cured. b) when the patient refuses recommended investigations or treatment c) when the patient manifests a hostile or irreverent attitude.

*The correct answer envisaged: „When the patient manifests a hostile or irreverent attitude.” (c)*

Self-assessment:

Question no. 11: „Have you performed medical acts that could be considered a law violation, in the past three years?” Proposed answer options: a) yes, b) no.
